# The mediating role of rumination in the relationship between negative cognitive styles and depression among pregnant women in Guangzhou, China

**DOI:** 10.3389/fpsyt.2024.1499061

**Published:** 2024-12-09

**Authors:** Min Liang, Yu Chen, Yan Liu, Ribo Xiong

**Affiliations:** ^1^ Department of Gynecology &Obstetrics, The Third Affiliated Hospital of Southern Medical University, Guangzhou, China; ^2^ School of Nursing, Southern Medical University, Guangzhou, China; ^3^ Department of Rehabilitation, The Seventh Affiliated Hospital, Southern Medical University, Foshan, China

**Keywords:** rumination, negative cognitive styles, depression, pregnant, mediation

## Abstract

**Backgrounds:**

Negative cognitive styles (NCSs) have been identified as risk factor for the onset of depression. However, little empirical evidence is available to support its role in psychological disorders in the perinatal period. Moreover, less is known about the underlying mechanism in the relation between NCSs and depression in pregnant women. The purpose of this study was to examine the mediation effect of rumination on the relationship between NCSs and antenatal depression (AD). Specifically, the mediation effects of two subtypes of rumination were tested.

**Methods:**

A cross-sectional study was conducted from February to May 2023 using anonymous online questionnaire among women in their third trimester of pregnancy in the antenatal care clinic of a tertiary hospital. The Edinburgh Postnatal Depression Scale was used to screen antenatal depression. Attributional Style Questionnaire and Ruminative Responses Scale were employed to assess NCSs and rumination respectively. Correlational analysis of the associations between NCSs, rumination, and AD was conducted. Bootstrap mediation analysis and multiple mediation models were applied to investigate whether rumination, and its brooding and reflection components would mediate the relationship between NCSs and AD.

**Results:**

NCSs had a significant positive effect on depression in pregnant women (c=1.45, SE=0.03, *p*<0.001, 95%CI: 0.92∼1.70). Rumination mediated the relationship between NCSs and depression in pregnant women (point estimate=0.41, 95%CI: 0.13∼0.79, effect size=0.22, K^2^ = 0.19). Multiple mediation analysis revealed that brooding, instead of reflection, mediated the relationship between NCSs and depression in pregnant women (point estimate=0.41, 95%CI: 0.15∼0.78).

**Conclusion:**

This study provided novel evidence for the role of rumination, specifically its brooding subtype, in shaping the link between NCSs and depression in pregnant women, highlighting potentially useful targets for interventions aimed at preventing the onset of AD.

## Introduction

Antenatal depression (AD) (1), manifested by persistent low mood, feelings of guilt or worthlessness, lack of enthusiasm, and feeling isolated or isolated, is one of the most common mental health problems during pregnancy with a prevalence ranging from 11.9% to 19.7%. Evidence has shown (2) that AD has adverse effects on obstetric and neonatal outcomes, such as premature delivery, cesarean section, low birth weight, postpartum depression. Studies also suggest (3, 4) the transmission of psychopathology from a mother to her offspring. Therefore, antenatal period is a good opportunity for health professionals to prevent and control.

Recent research has highlighted the important role of cognitive processes in the onset and maintenance of psychological disorder in the perinatal period (5). Cognitive vulnerability is often thought of as a negative cognitive style (NCS) (6), referred to a tendency to respond to situations with negative appraisals about one’s self as well as the causes and consequences of the situation. A number of research have clearly indicated an association between a negative cognitive style and depressive symptoms both in children and adolescents (7, 8). Nevertheless, little empirical evidence is available to support the role of negative cognitive styles in psychological disorder in either antenatal or postnatal periods. Given that this period is characterized by unique episodes, such as hormonal and body shape changes, insomnia that could potentially interact with cognitive processes, pregnant women may display a more NCS which predicts the subsequent development of depressive symptoms as their underlying negative thinking makes it very difficult to exit the self-regulatory cycle. Moreover, from a clinical perspective, NCS is a modifiable risk factor which contributes to depression is exciting and raises the possibility that interventions may also have utility in preventing the emergence of depression. Therefore, it is it is imperative to develop a better understanding of the role of NCS in the perinatal context.

Rumination (9), a key cognitive feature of depression, attracted considerable interest in the perinatal context recently and has been identified as an important risk factor for depression in the perinatal period. In a longitudinal study, Barnum proposed (10) that individuals who engage in rumination tend to have more depressive symptoms from the third trimester to 8 weeks postpartum. One possible mechanism (11) through which rumination contributes to depression is by enhancing the combined effects of cognitive biases and cognitive control deficits. During pregnancy, cognition may be biased in favour of negative information, which could be triggered by an intrusive negative thought, or an everyday stressor. Following this trigger, these biases are therefore strengthened via rehearsal and thus creating a vicious cycle of cognitive-affective processing. Findings from clinical samples (12) have also indicated the detrimental effects of rumination on cognitive correlates of depression, such as pervasively negative thinking and overgeneral autobiographical memory. On the other hand, rumination was found to be a key transdiagnostic mechanism linking vulnerability factors to depression (13).

It is recognized that negative cognitive styles, rumination and depression are interrelated. Whilst rumination may play a mediating role between cognitive processes and depression, it has been much less characterized in the context of AD. Furthermore, AD is always related with (2) several socio-demographic and psychosocial factors, such as internal immigration status, low socio-economic status and low social support. These factors may have a profound effect on cognitive processes, which contribute to an increased antenatal depressive level. How this socio-cultural background affects the mental health of pregnant women continuously attract researcher’s interest. China, the world’s most populous nation, is being challenged by the unprecedented internal migration. Since the Reform and Opening up in 1980s, young people have been moving to coastal and urban areas to seek better livelihood. Guangzhou, where this study was conducted, is one of the largest migrant-concentrated metropolis in China.With nearly 50% of it’s total population being migrated and 50% of it’s migrants being female, Guangzhou provided a natural environment where the maladjustment such as depression and anxiety can be observed among immigrant women. We hypothesized negative cognitive styles would have the positive correlations relative to AD. We further predicted that rumination would mediate this association.

## Method

### Study setting

The study was carried out in the antenatal care clinic of the Third Affiliated Hospital of Southern Medical University, in Guangzhou of south China. Guangzhou had a population of more than 16 million. More than 50% of its total population were migrants and among migrants nearly 50% were female.The number of women attending antenatal care in this hospital reached approximately 5000 per year and the average number of childbirths was 300∼400 per month. In China, usual antenatal care includes ten visits throughout different gestational weeks.

### Participants

Women in their third trimester who underwent prenatal checkups in the antenatal care clinic during the period from February to May 2023 were offered the opportunity to participate in the study. Inclusion criteria were as follows: 1) Chinese nationality; 2) being pregnant in their third trimester; 3) providing informed consent. Women with previous diagnosis of psychiatric disorders or maternal/fetal diseases were excluded.

Of the 462 women who were invited, 451 women met the inclusion criteria. Among those eligible women, 19 did not complete the questionnaire. Eventually, we included 432 respondents.

### Data collection

To collect data for the present study, first, potential respondents were invited by three nurses. When they consented to the participation, an anonymous questionnaire through an online survey platform (‘SurveyStar’, Changsha Ranxing Science and Technology, Shanghai, China) was distributed to them. Anonymous submission has been set up on the ‘SurveyStar’ to ensure confidentiality. Once the questionnaire was completed and submitted, research assistants (three nurses and six post-graduates) can collect the data using the computer version of ‘SurveyStar’ on the desktop computer or laptop.Small gifts (sanitary towel) were given to them when they submitted the questionnaire. The research assistants were available throughout the survey to assist with participants, and regular supervision and feedback were carried out daily by the principal researcher during the data collection period.

We recruited 136 women with antenatal depression allowing detection of a mediated effect with a power of 0.86 calculated by Gpower software.

### Measures

#### Attributional Style Questionnaire

Negative cognitive styles was assessed with Attributional Style Questionnaire (ASQ) (14), which is a recommended assessment tool for measuring cognitive vulnerability because of its good psychometric properties.This self-report measure consists of six hypothetical positive and six hypothetical negative events that relate to three dimensions of internality, stability, and globality. Items of six negative events were rated on a 7-point Likert scale, ranging from 3 to 21. A translation and back translation procedure was used to develop the Chinese version of the ASQ. The internal consistency proved to be satisfactory, with Cronbach’s alpha 0.82 for the current study.

#### Ruminative Responses Scale

The RRS was used to assess how participants tend to respond to their depressive mood (15). It contains 22 items and responses are scored on 4-point Likert scales, resulting in a possible range of scores from 22 to 88. A recent study demonstrated (16) that the RRS comprises two distinct factors, brooding and reflection, each with five items that were not confounded with depressive content. A translation and back translation procedure was used to develop the Chinese version of the RRS. In the present study, the coefficient alphas for the brooding and reflection subscales were 0.65 and 0.70, respectively.

#### Edinburgh Postnatal Depression Scale

The Edinburgh Postnatal Depression Scale (EPDS) (17) is a 10 item self-report questionnaire of depressive symptoms in pregnancy and the postpartum period. Although originally developed for postpartum depression, the EPDS has also been validated for antenatal depression screening (18). A cutoff score of 13 or higher exhibits good sensitivity (86%) and specificity (78%) in detecting depressive diagnoses in antenatal women.The Chinese version of the EPDS had satisfactory reliability and validity, with a Cronbach’α of 0.96 and correlation coefficient of 0.79.

### Statistical analysis

SPSS20.0 statistical package was used for data analysis. In the first step, correlational analysis of the associations between negative cognitive styles, rumination, and antenatal depression was conducted. A *p*-value<0.05 was identified statistically significant. In the second step, bootstrap mediation analysis was applied to investigate whether rumination, and more specifically its brooding and reflection components would mediate the relationship between negative cognitive styles and depression in pregnant women. Bootstrapping method for 5000 samples was utilized for coefficient and indirect estimation. More precisely, multiple mediation models via two facets of rumination (brooding and reflection) were constructed, mediating the association of negative cognitive styles and antenatal depression. In addition to *p* values, asymmetric confidence intervals were used to verify the effect for significance. If zero was not included in the 95% bias-corrected confidence intervals (CIs), the effect was considered statistically significant. Standardized estimates and K^2^ were reported as index of effect size.

### Ethical considerations

Ethics approval was obtained from the Research Ethics Board of The Third Affiliated Hospital of Southern Medical University (No.1001841).

## Results

The age of participants ranged from 22 to 38 years. The educational level of participants ranged from 9 to 18 years. Parity ranged from 0 to 3. 54.6% of the participants were internal migrants. Out of the 432 women who were enrolled in the assessment, we have reported that 136 women were screened positive for AD with EPDS at a cut-off point of 13, thus resulting in a prevalence of 31.4%. Of the women with AD, 60% were immigrants.

### Correlations between NCSs, EPDS, Rumination, brooding and reflection

**Table 1** presents correlations, mean scores and SDs for each of the five variables. As predicted, rumination was positively associated with both NCSs (*r=*0.71, *p*<0.001) and EPDS (*r=*0.59, *p*<0.001). Brooding was positively associated with NCSs (*r=*0.69, *p*<0.001) and EPDS (*r=*0.56, *p*<0.001). Reflection was positively associated with NCSs (*r=*0.35, *p*<0.001) and EPDS (*r=*0.38, *p*<0.01). Yet these two rumination subscales were uncorrelated.

**Table 1:** Descriptive statistics and correlations of measured variables and subscales (N=136).

Variable	Mean	SD	1	2	3	4	5
1. EPDS	22.7	6.3	1	0.67^**^	0.59^**^	0.56^**^	0.38^*^
2. NCSs	15.4	4.8		1	0.71^**^	0.69^**^	0.35^*^
3. Rumination	49.6	11.9			1	0.66^**^	0.60^**^
4. Brooding	13.3	2.6				1	0.22
5. Reflection	11.5	3.7					1

EPDS, Edinburgh Postnatal Depression Scale; NCSs, Negative cognitive styles. *P < 0.01; **P < 0.001.

### Mediation model for the relationship between NCSs and antenatal depression

**Figure 1:**
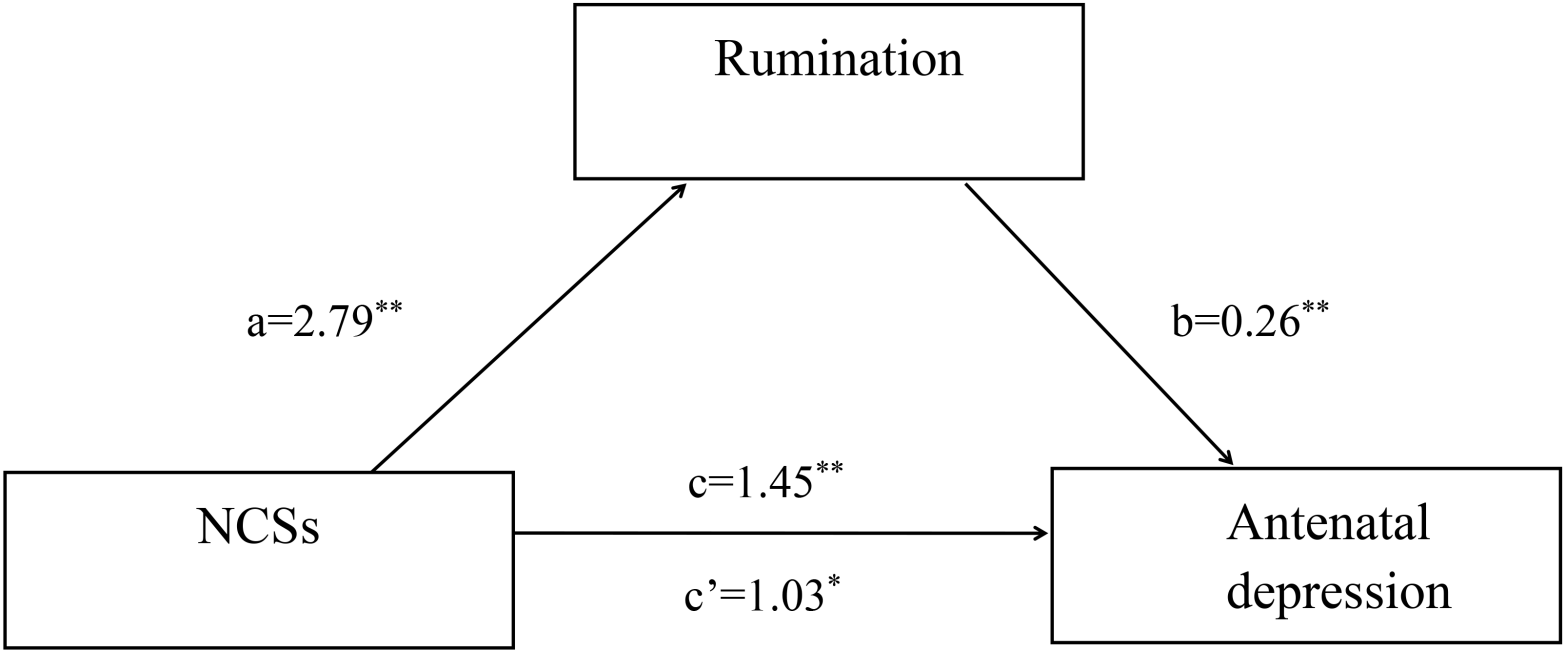
Simple mediation model. Unstandardized path coefficients indicated above. c=total effect; c’=direct effect. ^*^ *P* < 0.01; ^**^ *P* < 0.001.

**Figure 2:**
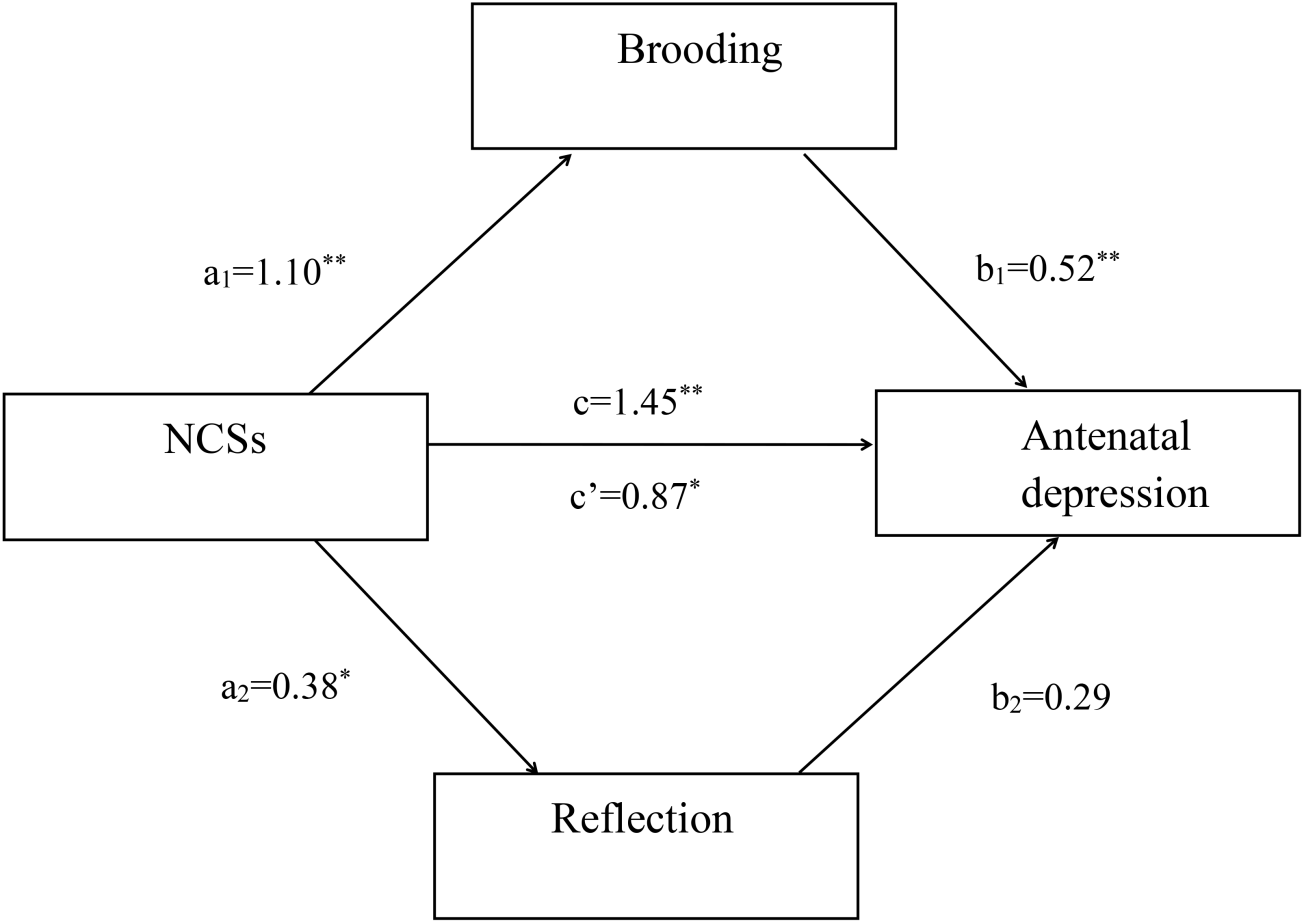
Multiple mediation model. Unstandardized path coefficients indicated above. c = total effect; c’ = direct effect. *P < 0.01; **P < 0.001.

Simple mediation analysis revealed that NCSs had a significant positive total effect on antenatal depression (c=1.45, SE=0.03, *p*<0.001, 95%CI: 0.92∼1.70). The direct effect from NCSs to antenatal depression remained significant (c’=1.03, SE=0.11, *p*<0.01, 95%CI: 0.65∼1.48). As expected, NCSs had a significant positive effect on antenatal depression via rumination (point estimate=0.41, 95%CI: 0.13∼0.79) with an effect size of 0.22 (K^2^ = 0.19). According to Cohen’s conventions, the calculated effect size in this study suggested rumination mediated the relationship between NCSs and depression in pregnant women (**Figure 1**). Multiple mediation analysis revealed that NCSs had a significant positive total effect on antenatal depression (c=1.45, SE=0.16, *p*<0.001, 95%CI: 0.79∼1.88). The direct effect from NCSs to antenatal depression remained significant (c’=0.87, SE=0.18, *p*<0.01, 95%CI: 0.45∼1.53). The total indirect effect of NCSs on antenatal depression through brooding and reflection was significant (point estimate=0.46, 95%CI: 0.20∼0.83) with an effect size of 0.26. The specific indirect effect of NCSs on antenatal depression via brooding was also significant (point estimate=0.41, 95%CI: 0.15∼0.78), whereas reflection was not found to mediate this relationship (point estimate=0.08, 95%CI: -0.03∼0.27). According to Cohen’s conventions, the calculated effect size in this study suggested brooding mediated the relationship between NCSs and depression in pregnant women (**Figure 2**).

### Reverse mediation model for the relationship between NCSs and antenatal depression

Reverse mediation analysis was utilized to examine whether NCSs mediated the relationship between rumination and antenatal depression. We also explored the mediating effects of rumination on the relationship between antenatal depression and NCSs. The results showed that NCSs did not mediate the relationship between rumination and antenatal depression (point estimate=0.13, 95%CI: -0.04∼0.61). Similarly, rumination did not show a significant indirect effect on the relationship between antenatal depression and NCSs (point estimate=0.17, 95%CI: -0.06∼0.48). Because zero is included in the 95% confidence interval, it can be concluded that the mediation effects of rumination is directional specifically.

## Discussion

Although NCSs is a well-established risk factor for depression, little is known about its role during the antenatal period. One possible mechanism (11) through which NCS may have an impact on depression is by enhancing the repetitive thinking on negative cognition. Findings from experimental studies (12) have provided evidence for the role of rumination as a common proximal mechanism relating depressive risk factors to depression. This is one of the first studies examining the mediation effects of rumination on the relationship between NCSs and depression in pregnant women. Specifically, the mediation effects of brooding and reflection components of rumination were also tested.

As hypothesized, the present study revealed NCSs was significantly correlated with global rumination. The relationship between NCSs and depression was partially mediated by rumination in pregnant women. This finding is consistent with previous studies of adults (14, 19) in which a higher level of NCSs was associated with later increases in rumination. A variety of cognitive and neurobiological mechanisms has been proposed to underlie this relationship. On the one hand, from the perspective of control theory (20), induction of NCSs can create discrepancies between desired states and current state, which can increase self-focused rumination to reduce these discrepancies. Activation of NCSs may be particularly likely for expectant mother given that the antenatal period is characterized by tremendous, complex change and adjustment (e.g., in social roles, interpersonal relationships). Moreover, rumination on discrepancies following NCSs may persist because it focuses on the causes and/or negative consequences of the events, rather than on problem solving. This view was in line with research on rumination (14) which proposed cognitively vulnerable individuals were at higher risk for engaging in rumination as their underlying NCSs made it difficult to exit the self-regulatory cycle. On the other hand, hormonal changes that occur at different stages throughout this period do have an influence over increased emotional reactivity (21). This view was similar to results observed in another sampling study (22) documenting that women with premenstrual dysphoric disorder displayed a more NCS during the late luteal phase than a healthy control group. Through its receptors, both oestrogen and progesterone have a great influence over the function of the hypothalamic-pituitary-adrenal axis (22), thus enhancing the reactivity to stress. We proposed that NCSs were awakened and strengthened during pregnancy, contributing to increased engagement in rumination. Thus, the findings suggest pregnant women with higher levels of NCSs tended to engage in rumination, which in turn predicted antenatal depression.

As expected, the present study showed NCSs was significantly correlated with brooding. Brooding mediated the relationship between NCSs and depression in pregnant women. This finding was in conjunction with a previous study (11) that found brooding was strongly associated with greater depression both concurrently and longitudinally. Moreover, brooding was found to mediate the effects of social support, negative thinking and neuroticism on depression (14). Accumulating research (11) has identified the presence of two subtypes of rumination with distinctive neural basis and functional properties. Brooding represents a tendency toward moody pondering and reflects a passive comparison of one’s current situation with some unachieved standard. We speculate that brooding, as a maladaptive element of rumination, could trigger the underlying negative schemas frequently and habitually, and hence intensify the effects of the negative thinking on depression. The present finding further specify brooding as a detrimental component which may linking depressive risk factors to perinatal depression.

In addition, the results indicated reflection, another subtype of rumination, did not mediate the relationship between NCSs and depression among pregnant women. This result suggested that reflection is an adaptive component of rumination. It may be the case that individuals who engaged in reflection could find a strategy, thus lower depressive symptoms with adaptive coping responses.

It’s noteworthy that two thirds of women with AD in this study are internal migrants. Due to the fact that the utility and allocation of public resources is based on household registration (“Hukou”) policy, immigrants do not have the same rights and benefits as local registered residents in a variety of areas, such as health care, social services, offspring education and housing. In addition, immigrant women could confront challenges in a new host society, such as adjusting to a new language, unfamiliar laws, different norms for social interaction and lifestyle changes, isolation from their families. If some events, which challenge their core beliefs, take place, the stream of negative thoughts may run through their minds, and it will cast a negative interpretation of the events. This negative interpretation may increase the stress level and cause depressive symptoms.

A few clinical implications can be drawn from this study to improve the psychological well-being of pregnant women. First, rumination-focused cognitive-behavioral therapy should be applied. The goal of this intervention is help individuals identify their ruminative thoughts and help them to focus on their problems purposefully and engage in active and effective problem solving. Second, given that a majority of expectant mothers in this study is internal immigrants, it is important to involve social integration programs in cognitive-behavioral therapy.

Several limitations should be borne in mind. First, a cross-sectional design did not allow us to infer a causal relationship about the results or investigate the dynamic process. Therefore, longitudinal research should be conducted to continue to test our mediated moderation model in the future. Second, our sample consisted of women identifying as predominantly migration status. As underserved population experience particular barriers and stress, which would likely enhance ruminative process and mental health symptoms, future research is needed to incorporate larger number of women from different backgrounds. Third, since EPDS was employed as a screening tool to define AD instead of a clinician-administered structured diagnostic interview, caution should be exercised before generalizing the present results to clinical population. By including a group of psychiatric patients, we were able to test the mediation model in the participants with a wide range of depressive symptoms. Fourth, variables were measured at one time point, future research should employ a prospective design to establish the mediation with more certainty.

## Conclusion

Global rumination partly mediated the relationship between NCSs and depression among pregnant women. Furthermore, the mediating role of rumination in this relationship is mainly due to the brooding subtype of rumination. The current finding suggests psychological interventions focused on disengaging from brooding, as well as active cognitive problem solving.

## Data Availability

The raw data supporting the conclusions of this article will be made available by the authors, without undue reservation.
